# Antenatal management of fetal neurosurgical diseases

**DOI:** 10.1007/s00381-017-3442-x

**Published:** 2017-05-29

**Authors:** Sergio Cavalheiro, Marcos Devanir Silva da Costa, Jardel Nicacio Mendonça, Patricia Alesssandra Dastoli, Italo Capraro Suriano, Mauricio Mendes Barbosa, Antonio Fernandes Moron

**Affiliations:** 1 Department of Neurosurgery, Federal University of Sao Paulo, Rua Botucatu, 591, conj 41, Sao Paulo, SP CEP: 04023-062 Brazil; 2 Department of Gynecology and Obstetrics, Federal University of Sao Paulo, Rua Botucatu, 591, conj 41, Sao Paulo, SP CEP: 04023-062 Brazil

**Keywords:** Hydrocephalus, Myelomeningocele, Encephalocele, Cephalocentesis, Ventricle-amniotic shunt

## Abstract

The advance in the imaging tools during the pregnancy (ultrasound and magnetic resonance) allowed the early diagnose of many fetal diseases, including the neurological conditions. This progress brought the neurosurgeons the possibility to propose treatments even before birth. Myelomeningocele is the most recognized disease that can be treated during pregnancy with a high rate of success. Additionally, this field can be extended to other conditions such as hydrocephalus and encephaloceles. However, each one of these diseases has nuances in the diagnostic evaluation that should fit the requirements to perform the fetal procedure and overbalance the benefits to the patients. In this article, the authors aim to review the neurosurgical aspects of the antenatal management of neurosurgical conditions based on the experience of a pediatric neurosurgery center.

## Introduction

In the twentieth century, fetal neurosurgical diseases were one of the main causes of maternal death. Charpentier (1887) described 200 cases of fetal hydrocephalus that accounted for 40 (20%) maternal deaths, mostly due to uterine ruptures [1]. The first fetal therapeutic procedure was performed by Liley (1963) by the injection of blood into the peritoneal cavity in a 32-week fetus affected by Rhesus (Rh) isoimmunization. The fetus showed a good outcome, and this case was the initial milestone for fetal surgery. The so-called fetal neurosurgery was not introduced until 3 years later when Barke et al. (1966) confirmed the diagnosis of fetal hydrocephalus through gas ventriculography. This non-therapeutic diagnostic procedure marks the beginning of fetal neurosurgical procedures [2]. Kellner et al. (1980) determined the thickness of the cortical mantle in a hydrocephalic fetus using fetal ventriculography and performed a transabdominal cephalocentesis during childbirth to avoid dystocia [3].

The first therapeutic neurosurgical procedures were carried out by Birnholz and Frigoletto (1981), who used repeated cephalocentesis in a hydrocephalic 25-week fetus, performing 6 ultrasound-guided transabdominal punctures [4], and by Clewel et al. (1982), who described the first ventriculoamniotic shunting of a 24-week-old fetus, which was effective until the 32nd week of gestation [5]. Maning et al. (1986) consolidated the results of fetal surgeries recorded in the “International Fetal Surgery Registry” and how these were considered poor; they contributed towards the discontinuation of fetal hydrocephalus treatment [6]. Fetal neurosurgery did not advance until 1999 when Tulipan et al. demonstrated a reversal of Chiari II malformation after the intrauterine correction of fetal myelomeningocele using open fetal surgery, when performed before the 26th week of gestation [7]. In 2003, Cavalheiro et al. performed the first fetal endoscopic third ventriculostomy for the treatment of hydrocephalus by aqueduct stenosis in a fetus at 26 weeks of gestation [8]. In 2011, Adzick et al. published the results of an antenatal surgery to correct myelomeningocele, called the Management of Myelomeningocele Study (MOMS), and inaugurated a phase in which fetal surgery for myelomeningocele became superior to postnatal surgery [9]. The objective of this work is to review and discuss the therapeutic possibilities of neurosurgical diseases during the fetal period.

## Fetal hydrocephalus

Fetal hydrocephalus is a complex and multifactorial disease. The period during which the fetus suffers the insult culminating in hydrocephalus is one of the most important factors to consider when establishing a prenatal prognosis. Fetal hydrocephalus diagnosed in the last trimester of the gestation usually have a better outcome when compared with those that are diagnosed in the first and second trimester of the pregnancy. A similar behavior can be seen in the obstructive hydrocephalus that has a better outcome when compared with communicating hydrocephalus. Other malformations associated with hydrocephalus may occur in up to 75% of cases, providing a strong influence on the prognosis of fetuses affected by this disease.

One of the key points of prenatal diagnosis is the differentiation between fetal hydrocephalus and non-hypertensive ventriculomegaly. The former is eligible for intrauterine treatment with good results. The latter, however, may result in either a favorable or catastrophic outcome through a destructive etiology, as seen in viral infections such as the Zika virus. Therefore, non-hypertensive ventriculomegalies are typically not eligible for intrauterine treatment [10].

Institutions around the world interrupt the pregnancy when they face a case of fetal hydrocephalus based on the unpredictable prognosis of the patient. However, in many countries, the interruption of the pregnancy is forbidden or it is just allowed in cases that the mothers are at risk or in cases that the mothers were raped. This scenario justifies the development of fetal neurosurgery centers capable of creating and improving the techniques for the hydrocephalus treatment during the intrauterine life and minimize the harmful outcomes of this disease.

In 1981, Jeanty et al. [11] described a relationship between the lateral ventricle and the cerebral hemi-hemisphere (LV/CHH) based on the study of 200 normal fetuses. The graphical representation of this relationship permits ventriculomegaly to be diagnosed at an early gestational age, much earlier than any increase in the biparietal diameter. In many cases, the cranial circumference and the biparietal diameter only increase near the end of the gestational period. Huge ventriculomegaly often occurs without any increase in the cephalic perimeter.

During the fetal period, an atrial width <10 mm is considered normal; between 10 and 15 mm, the ventriculomegaly is classified as mild to moderate, whereas a measurement >15 mm is graded as severe [12]. In the group with mild and moderate ventriculomegaly, only 14% develop progressive hydrocephalus, 57% are stable, and in 29% regression occurs spontaneously [13].

Another imaging method contributing to antenatal practice is fetal magnetic resonance imaging (MRI). However, MRI is subject to artifacts due to movements of the fetus. Multiple reports have confirmed that fetal MRI is a valuable and important adjunct to US in a multitude of fetal brain pathologies [14, 15].

Fetal MRI is better suited than ultrasonography when evaluating processes of maturation and neuronal myelination. It is also not affected by issues such as acoustic shadowing in the evaluation of the cerebral cortex as well as during the differentiation of brain tissues. High-resolution images and ultrafast single-shot T2-weighted or half-Fourier single-shot turbo spin-echo (HASTE) sequences allow the investigation to be performed without the need for sedation of the fetus, and are sufficiently precise to allow for analysis of the fetal anatomy.

With a view to enabling prognosis and possible intrauterine treatment, blood tests of both the mother and the fetus are necessary in addition to the hydrocephalus diagnosis. The analyses should include the study of congenital infections acquired during gestation as well as a study of the fetal karyotype. Cordocentesis should be performed as a matter of routine before any clinical decision regarding treatment. However, the association of fetal hydrocephalus with congenital infection or chromosomopathy contraindicates intrauterine therapeutic procedures.

Experimental studies in animal models have demonstrated that the earlier the treatment for fetal hydrocephalus is given, the greater efficacy it has. This type of result is not verified in daily clinical practice due to the large variety of diseases a fetus may present with. In the case of malformative hydrocephalus, many patients present with multiple associated malformations, which compromise a good outcome [16–19].

In fetal ventricular dilatation, it is very important to differentiate between ventriculomegaly and hydrocephalus. Ventriculomegaly may be the result of atrophy or hypoplasia of the central nervous system or malformation associated with agenesis of the corpus callosum, while in hydrocephalus, the ventriculomegaly is hypertensive. It can be very difficult to differentiate between the two conditions. In hydrocephalus, there is often a decrease in the subarachnoid space and dangling of the choroid plexus. The angle formed between the wall of the ventricle and the choroid plexus is increased. In ventriculomegaly, on the other hand, the subarachnoid space and choroid plexus are preserved [20]. Cavalheiro et al. [8] reported the results from 36 intrauterine-treated fetuses and found that all those that presented levels of intracranial pressure above 20 cm H_2_O had a better cognitive and motor development than those that presented low levels of intracranial pressure. However, not always reverting ventriculomegaly lead to a reversal of the devastating effects already caused by hydrocephalus and not even of those caused by the associated malformation.

A correct differentiation between the various etiologies of ventriculomegaly is essential to predict outcome and to guide the various treatment options which may start as early as the antenatal period [21]. It is well-known that an isolated mild to moderate ventriculomegaly is linked to an abnormal outcome in 10–20% of children, whereas ventriculomegaly with associated anomalies or as part of a complex syndrome is characterized by an abnormal outcome in up to 40–50% of children [22]. After several years of treating and monitoring fetuses with fetal hydrocephalus, we can say that when facing a case of evolving acute hydrocephalus without other associated malformations, intrauterine procedures can be beneficial.

The following requirements have been proposed to select patients eligible to fetal treatment:

1. Hydrocephaly should be diagnosed at an early stage of gestation
2. It should not be associated with other malformations
3. A karyotype study should be performed in all cases
4. Ventricular dilatation should be progressive
5. The treatment should be managed by a multidisciplinary team comprising specialists in perinatology, ultrasonography, obstetrics, neurosurgery, and genetics.

**Fig. 1:**
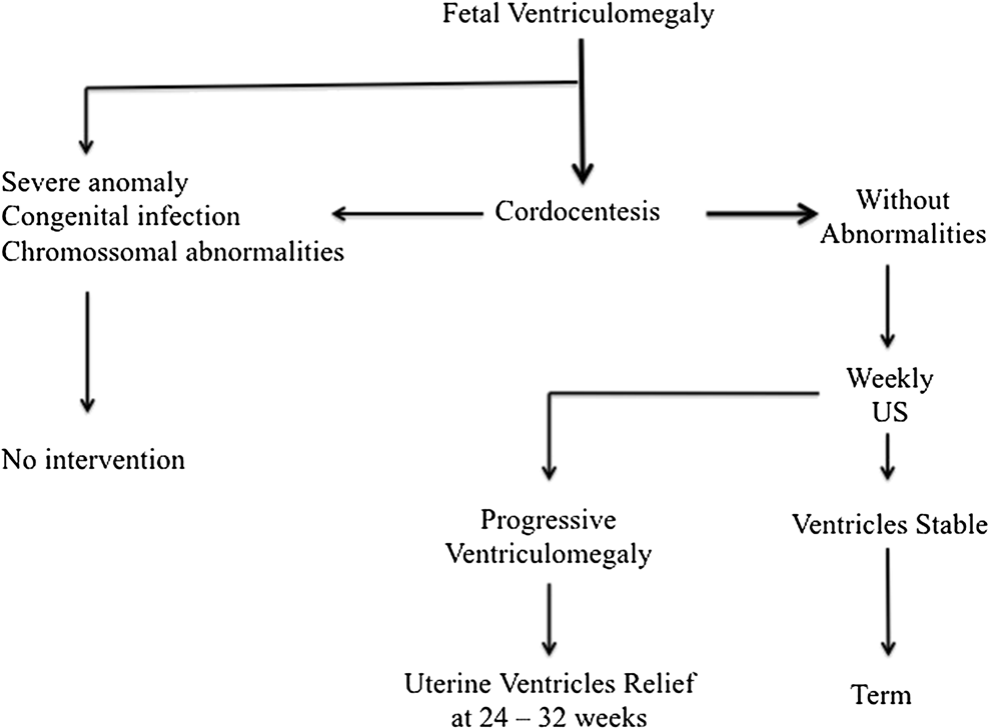
Algorithm for treatment of fetal hydrocephalus

The following algorithm (Fig. 1) is proposed for decision-making in fetal hydrocephalus at the Santa Joana Hospital and Maternity Center of Sao Paulo and the Neurosurgery and Obstetric Departments of Federal University of Sao Paulo.

**Fig. 2:**
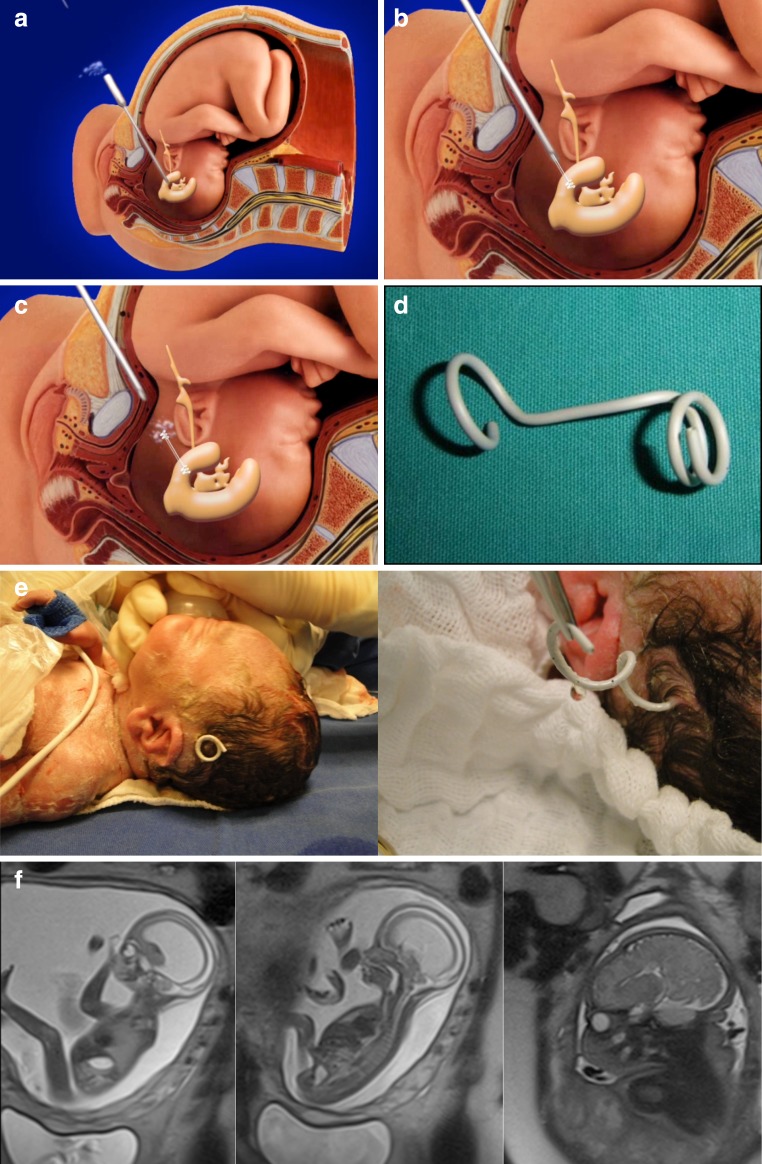
Scheme of ventriculoamniotic shunt placement. **a** Ultrasound-guided transabdominal puncture reaching the occipital horn of the lateral ventricle. **b** Catheter insertion and intraventricular portion released after partial removal of the trocar. **c** Complete removal of the trocar to release the catheter in the amniotic cavity and decrease hydrocephaly. **d** Double pigtail catheter. **e** Patient at birth exposing the ventriculoamniotic shunt used during the uterine life to treat a fetal hydrocephalus; in detail can be seen that the catheter was still working. **f** example of a fetal hydrocephalus due to aqueductal stenosis treated by ventriculoamniotic shunt. The pre-and post-MRIs show the reduction of the ventricular cavities after procedure

Although diagnoses of fetal malformations have improved during the last few years, the same improvement has not been seen in surgical techniques for the treatment of hydrocephalus. Surgical options include cephalocentesis, which consist of multiple ventricular punctures in order to remove liquor, and ventriculoamniotic shunts, which provide a fast decrease in the volume of the ventricular cavity but often migrate into either the ventricular or the uterine cavities (Fig. 2).

There are no ventricle peritoneal shunt systems that can be placed percutaneously and fixed on the skin of the fetus. On the other hand, fetal neuroendoscopy is feasible but technically very difficult because the fetus is seldom in a position that permits the surgeon to reach Kocher’s point and perform the procedure. Neuroendoscope procedures in fetuses and newborns are most complicated because the Lilequist membrane is too detached from the tuber cinereum at that age. For the procedure to be successful, it is necessary to open both membranes, which sometimes becomes very difficult in utero [23, 24].

Neuroendoscopic procedures have been attempted three times by our group, and it was effective in only one case. Furthermore, the literature is not unanimous over whether neuroendoscopic procedures are as effective in newborns as they are when performed after the first year of life, which causes doubt about the convenience of performing fetal endoscopic third ventriculostomy [25].

## Myelomeningocele

Open neural tube defects (ONTDs) are the most frequent malformations of the central nervous system (CNS). Myelomeningocele (MM) is the most severe open neural tube defect that is compatible with life. This closure disorder occurs in the third week of gestation, and biochemical, genetic, and environmental phenomena are involved in its genesis. Ninety percent of myelomeningocele patients develop hydrocephalus due to Chiari malformation type II and require ventricular shunts after birth [26]. Therefore, the main goal of the fetal surgery for myelomeningocele before 27 weeks of gestation is to reduce the ventricular shunt rates and revert the Chiari type II.

Tulipan and Bruner at the University of Vanderbilt in Nashville published the first report of an attempt to surgically treat fetal MM in 1997. These researchers described an endoscopic approach with implantation of a skin graft on the neural placode. The discouraging result (one patient died and another did not present neurological improvement) led to an abandonment of the endoscopic technique and the initiation of open surgeries [27, 28]. To date, the endoscopic procedures for intrauterine correction of MM are considered as experimental by the academic community. In 1999, Sutton et al. described an open surgery correction of fetal MM in ten patients between 22 and 25 weeks of gestation with encouraging results [29].

Furthermore, the Management of Myelomeningocele Study (MOMS) reported a 50% reduction in the rates of hydrocephalus and a two-level improvement in motor deficits when the correction of myelomeningocele is performed in utero [9], marking a new era in the treatment of myelomeningocele. Perhaps even further progress can be made in the treatment of myelomeningocele by a correction at an even earlier stage with the deployment of stem cells during the embryonic period. Yet another promising approach could be the development of more effective prophylactic methods than the use of folate before and after conception.

Much of the further content regards to our experiences from May 2011 to Dec. 2016 at the Santa Joana Hospital and Maternity Center of Sao Paulo, Brazil, and the Federal University of Sao Paulo (UNIFESP), Paulista Medical School after in utero treatment of 220 cases of myelomeningocele with open surgery.

### Antenatal diagnosis

The routine use of two-dimensional prenatal ultrasound (US2D) has facilitated the diagnosis of MM and rachischisis with increasing frequency. Ultrasonography is sufficient for an accurate diagnosis and has a detection sensitivity above 90%, which may or not be complemented with fetal magnetic resonance.

**Fig. 3:**
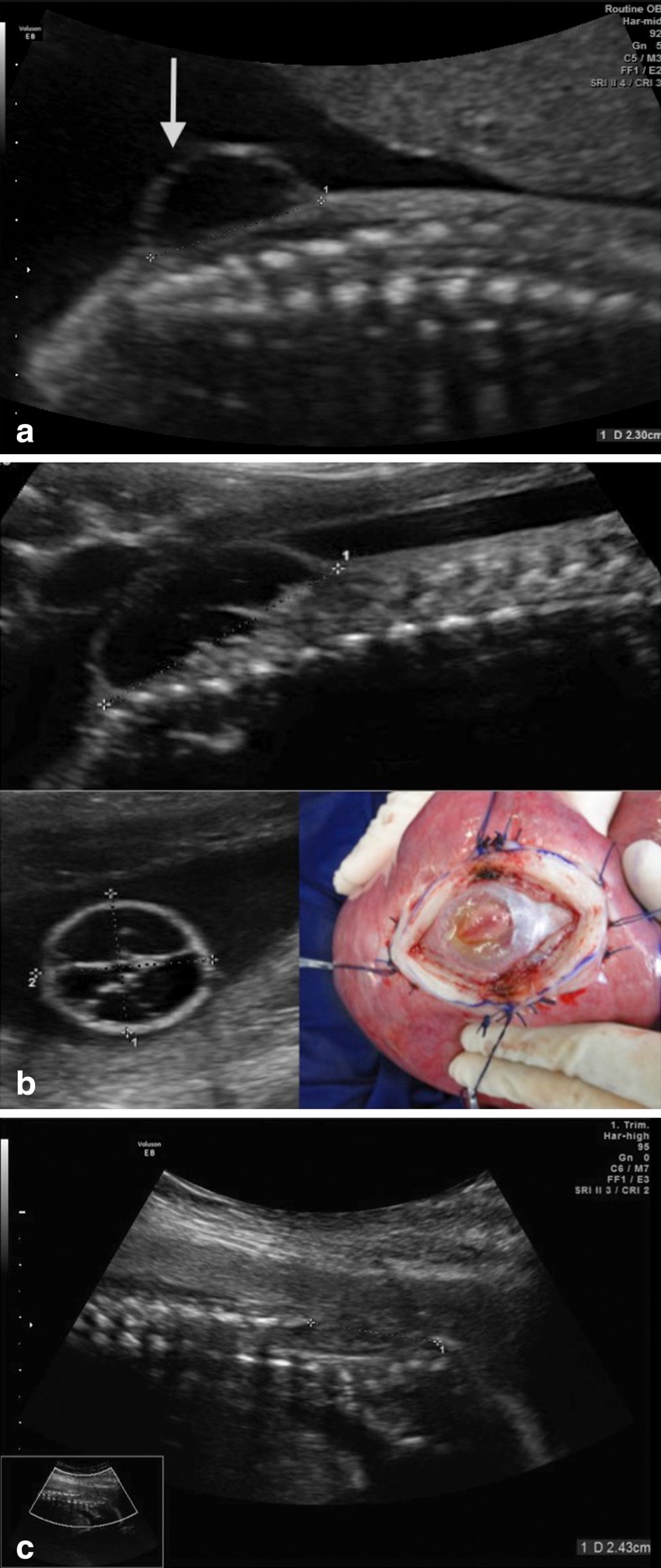
Two-dimensional ultrasound showing: **a** Meningocele. **b** Myelomeningocele with a neural tissue inside and the respective intraoperative view. **c** Rachischisis

**Fig. 4:**
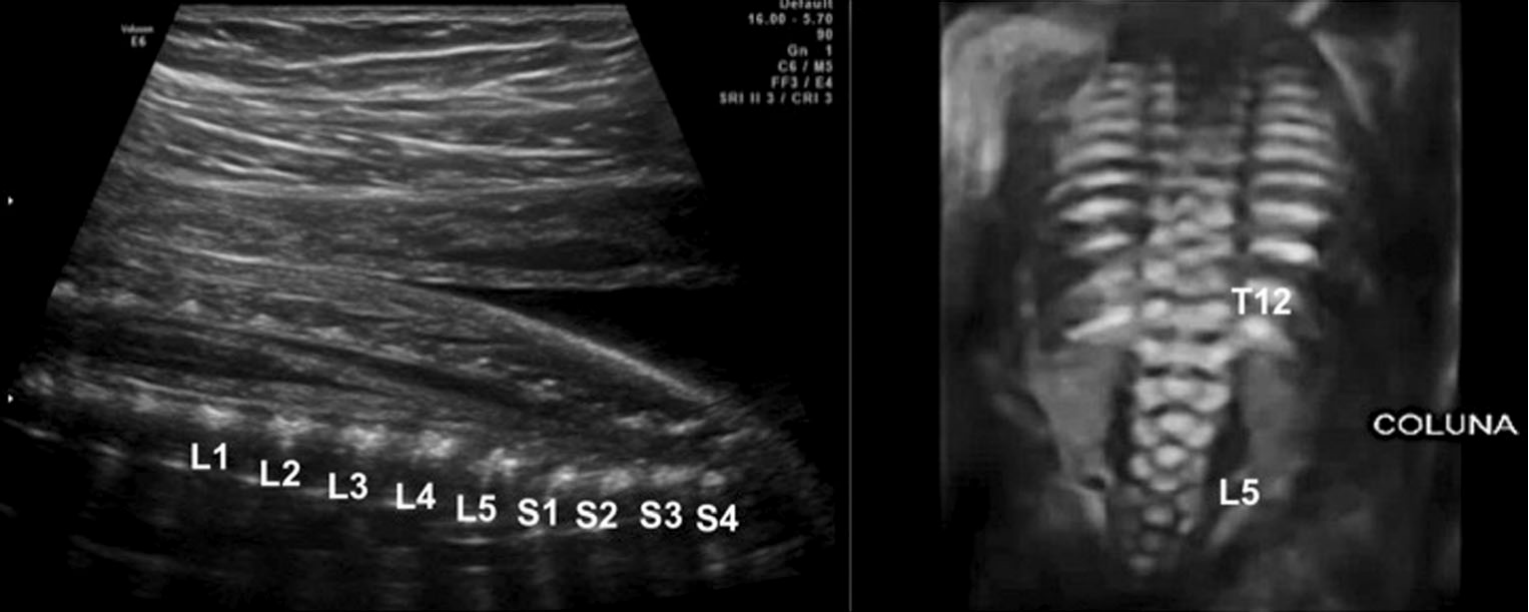
Two-dimensional ultrasonography. Counting of vertebral bodies by determining the 12th thoracic vertebra that corresponds to the last rib

**Fig. 5:**
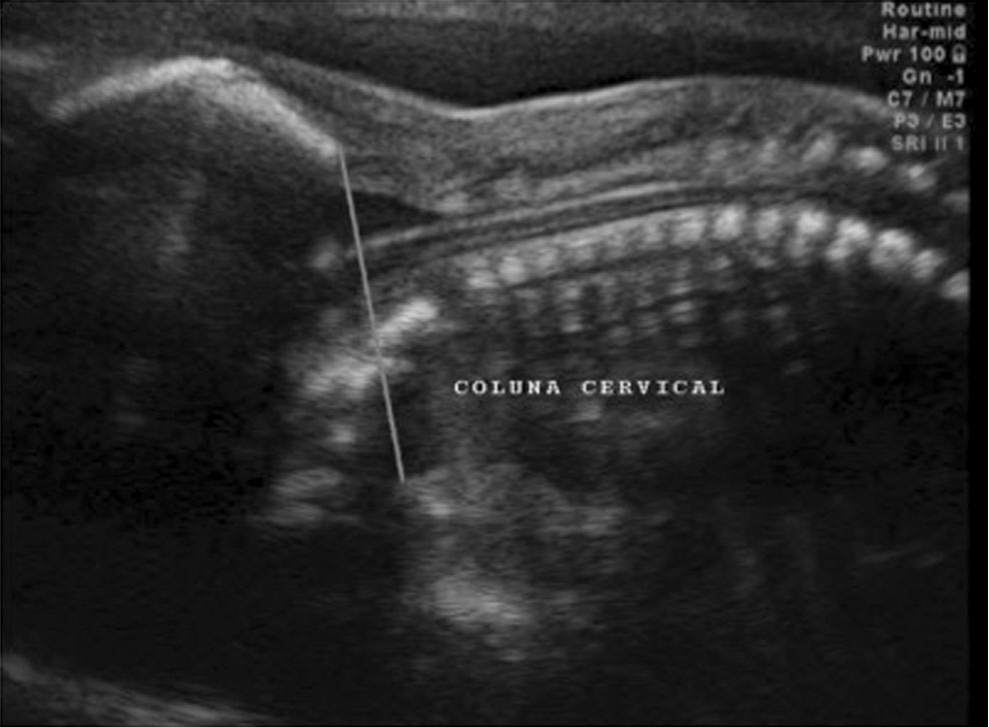
The occipitum-dens line described to evaluate the degree of the brainstem and tonsils herniation into to the cervical spine canal

US2D in association with three-dimensional ultrasonography allows for a detailed analysis of the characteristics of the lesion such as the following:

1. Type of spinal dysraphism. In cases of spina bifida, the posterior vertebral arches are not observed, with exteriorization of the meninges only (meningocele) (Fig. 3a), or meninges and nerve roots (myelomeningocele) (Fig. 3b), or only openings of vertebral bodies without the identification of the hernial sac (rachischisis) (Fig. 3c).
2. Anatomical level of the lesion (Fig. 4). The level of the lesion may be identified in most cases with T12 as a parameter and its count up to the level of the lesion.
3. Alterations of the curvature of the spine.
4. Associated malformations of the spinal canal, such as syringomyelia and diastematomyelia can be investigated.
5. Degree of herniation in the posterior fossa structures near the foramen magnum (Fig. 5). The occipitum-dens line, a new line cross between the cervical dens and the lower portion of the occipitum, can be used during an ultrasound to evaluate the normal level of the cerebellum, the brain stem in the posterior fossa, and the different degrees of cerebellar herniation in cases of Chiari type II [30].

**Fig. 6:**
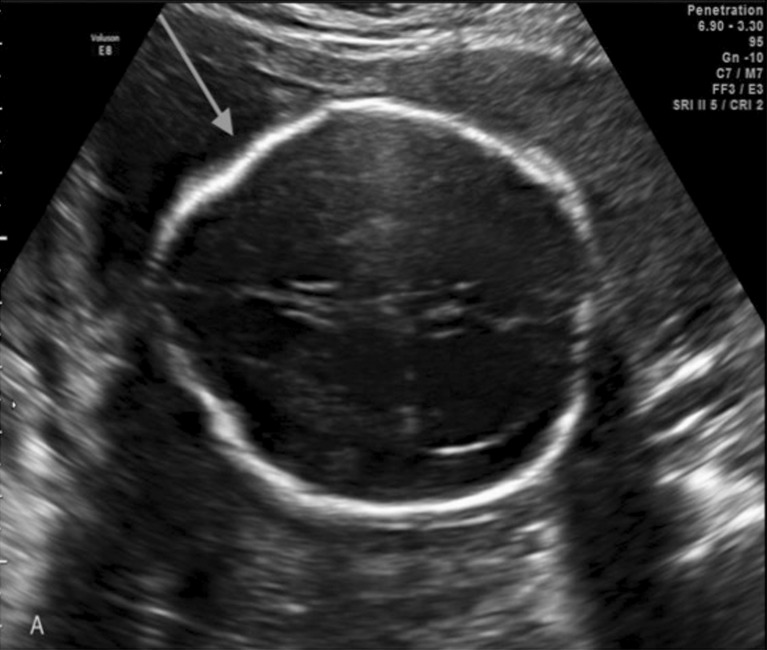
Two-dimensional ultrasonography. The “lemon sign”—the skull in a shape of a lemon due frontal scalloping bone caused by intracranial hypotension

**Fig. 7:**
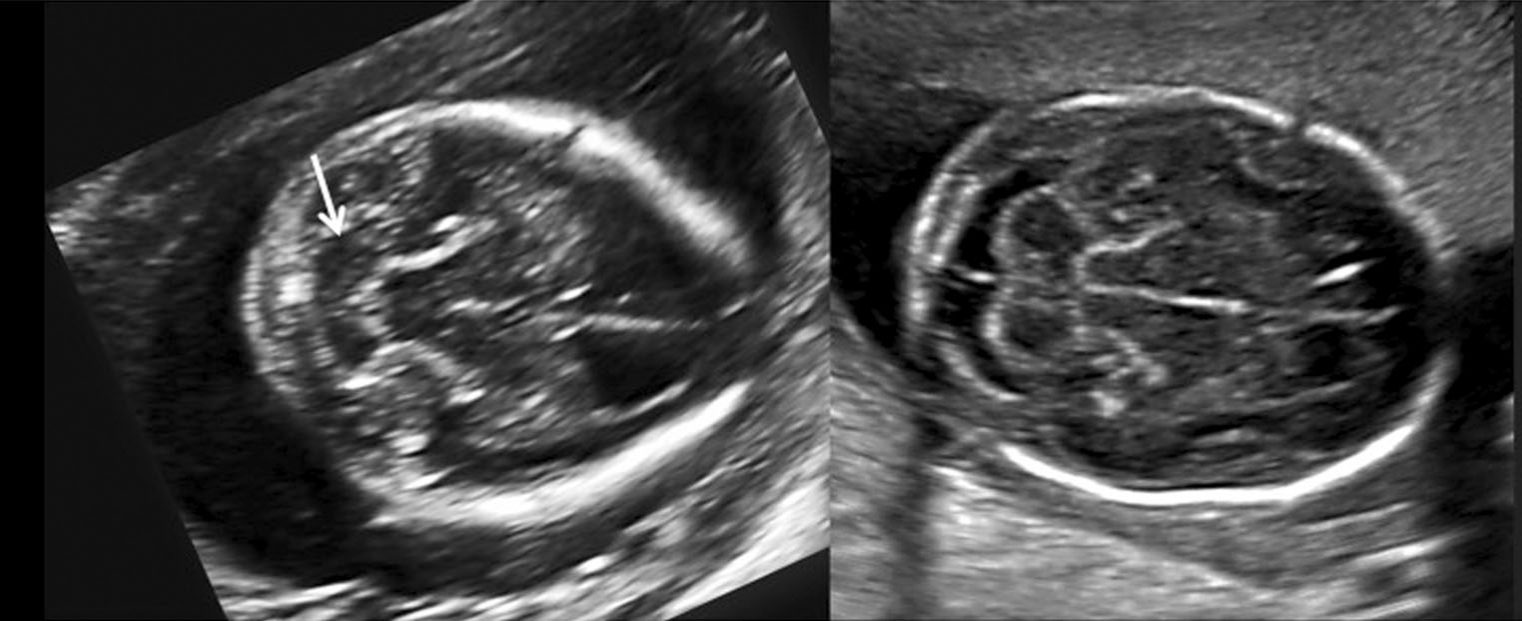
*Left image* shows the “banana sign” that represents the inversion of the cerebellum curvature (*arrow*). *Right image* shows a comparative normal posterior fossa and cerebellum and normal frontal shape

The presence of a cerebrospinal fluid leakage that is associated with a herniation of the cerebellar tonsil to the spinal canal determines variable degrees of intracranial hypotension that will be manifested by a small posterior fossa, an extended foramen magnum, a towering midbrain, and tectal breaking, in addition to a partial or total agenesis of the corpus callosum. Moreover, a direct ultrasonography evaluation of the fetal spine and cranial signs of spinal dysraphism can be evaluated from the 12th week of gestation and help in the diagnosis as follows:

1. The lemon sign can be used to describe the shape of the skull in the transverse plane and is present in many fetuses with MM. The lemon sign is characterized by the concavity of the frontal bone near the coronal suture as opposed to the convex configuration of the normal fetal skull [31, 32]. The lemon sign represents a scalloping of the frontal bones and features fetal intracranial hypotension (Fig. 6).
2. Ventriculomegaly can be assessed as a measure of the atria of the lateral ventricle that is greater than 10 mm [33] and is present in 70–90% of fetuses with an open SB. The characteristic pattern is colpocephaly with a dilatation of the occipital horns and preservation of the anterior horns, which is an aspect that can be related to the patterns of myelination.
3. The banana sign from the banana-shaped cerebellum can be used, which describes the echography findings in the axial plane (transcerebellar plan) arising from a herniation in the posterior fossa structures near the foramen magnum (cerebellum scotoma with diameter below the 10th percentile and obliteration of the cisterna magna [31, 34] (Fig. 7).

All three of the above cranial signals have a specificity of 99% but may also be present in normal fetuses of obese mothers [31].

Ultrasonography can be used to infer the degree of impairment of the lower limbs in fetuses with MM by identifying the presence of clubfoot and the degree of tropism of the lower limbs. It is possible to infer the amount of fat replacing the skeletal muscle in the presence of severe paralysis of the lower limbs. When actively evaluated, the movement of the lower limbs can demonstrate a good prognosis when compared to the presence of bilateral clubfeet. Notably, open dysraphism can produce involuntary movements of the fetus leading to a false diagnosis [35].

Fetal magnetic resonance is not superior to ultrasonography, but rather complementary. Nonetheless, it is uncertain whether the accuracy of diagnosis increases when the two procedures are performed before prenatal surgery. In some circumstances, performing both procedures is not possible, and many cases proceed with an operation after only an ultrasound exam. There are cases in which the two examinations are compelling, for example, when there are signs of dysraphism. However, the cranial aspects of the lemon and banana sign are not present. In these situations, lipomeningoceles or meningoceles may be present and must not be operated upon in utero. In our series of 262 cases of open antenatally diagnosed spinal dysraphism, we had 2 patients with diagnostic errors. One case involved a lipomeningocele, which was unnecessarily but successfully operated upon, and the other case involved a meningocele, which is a closed spinal dysraphism that presents no beneficial outcome. These cases are not included in this series. Another patient presented with diastematomyelia in addition to MM, which was also not diagnosed accurately.

The acquisitions of images are performed with ultrafast sequences to minimize the adverse effects of signs of maternal and fetal movements. T2-weighted (T2-W) images are enough for the majority of the information used for the diagnosis, including single-shot fast spin-echo (ssFSE) or half-Fourier acquisition single-shot turbo spin-echo (HASTE) sequences at minimal slice thickness (2–4 mm).

Ultrasonography provides more information to the obstetrician than to the neurosurgeon in fetal surgery for correction of MM. The obstetricians are much more familiar with ultrasonography while neurosurgeons are more familiar with MRI. MRI has a relatively low spatial resolution that is compensated by excellent soft-tissue contrast resolution between the cord and the surrounding cerebrospinal fluid, which probably accounts for the improved delineation of those structures [36]. MRI is also superior to ultrasonography for the detection of closed spinal dysraphisms such as lipomeningocele [37], as well as for the detection of other associated malformations such as the callosal dysgenesis/hypogenesis, periventricular nodular heterotopy, cerebellar dysplasia, syringohydromyelia, and diastematomyelia.

## Maternal aspects

The MOMS trial was initiated in 2003 and interrupted early in 2011 after the study of 183 cases (of a planned number of 200 cases) revealed a high efficacy of fetal surgery. The overall objective of the MOMS trial was to assess the results of intrauterine surgery between 19 and 25 weeks of pregnancy in comparison with postnatal surgery. The specific aim of the MOMS trial was to analyze the fetal or neonatal mortality rate and the incidence of ventriculoperitoneal shunts until 1 year of age. The secondary endpoint was an evaluation of cognitive and motor development at 30 months of age. The main inclusion criteria were pregnancy, MM from level T1 to S1, a gestational age between 19 and 25.6 weeks, a randomized and normal karyotype, and a maternal age above 18 years. The major exclusion criteria were fetal anomalies unrelated to MM, severe kyphosis, risk of premature birth (including short cervix and previous preterm delivery), and placental abruption. The study showed a statistically significant reduction in incidence of ventriculoperitoneal shunts (VPS) when compared to the prenatal group (40 versus 82%). Moreover, 42% of the prenatal group presented an independent gait at 30 months of life compared with 21% in the postnatal group (*p* < 0.001). Chiari malformation II was reversed in 36% of the prenatal group compared to only 4% of the postnatal group; the symptomatic Chiari II index was 6 and 22%, respectively. Fetal benefits should be evaluated from the perspective of increased maternal risk due to the increased incidence of complications such as premature rupture of membranes (46%), premature birth (79%), and the observed mean gestational age at birth of 34.1 weeks in the fetal surgery group compared with 37.3 weeks in the postnatal surgery group. In addition, a uterine scar from a hysterotomy resulted in various complications, including varying degrees of weakness of the uterine wall in 25% of the women at birth, 9% partial rupture, and 1% total rupture of the uterine scar [9].

Although the MOMS trial used strict inclusion and exclusion criteria for its study patients, in everyday practice, clinicians can be a little less rigid. Patients with controlled hypertension, diabetes, and some types of autoimmune diseases, such as systemic lupus erythematosus, can undergo the procedures.

**Fig. 8:**
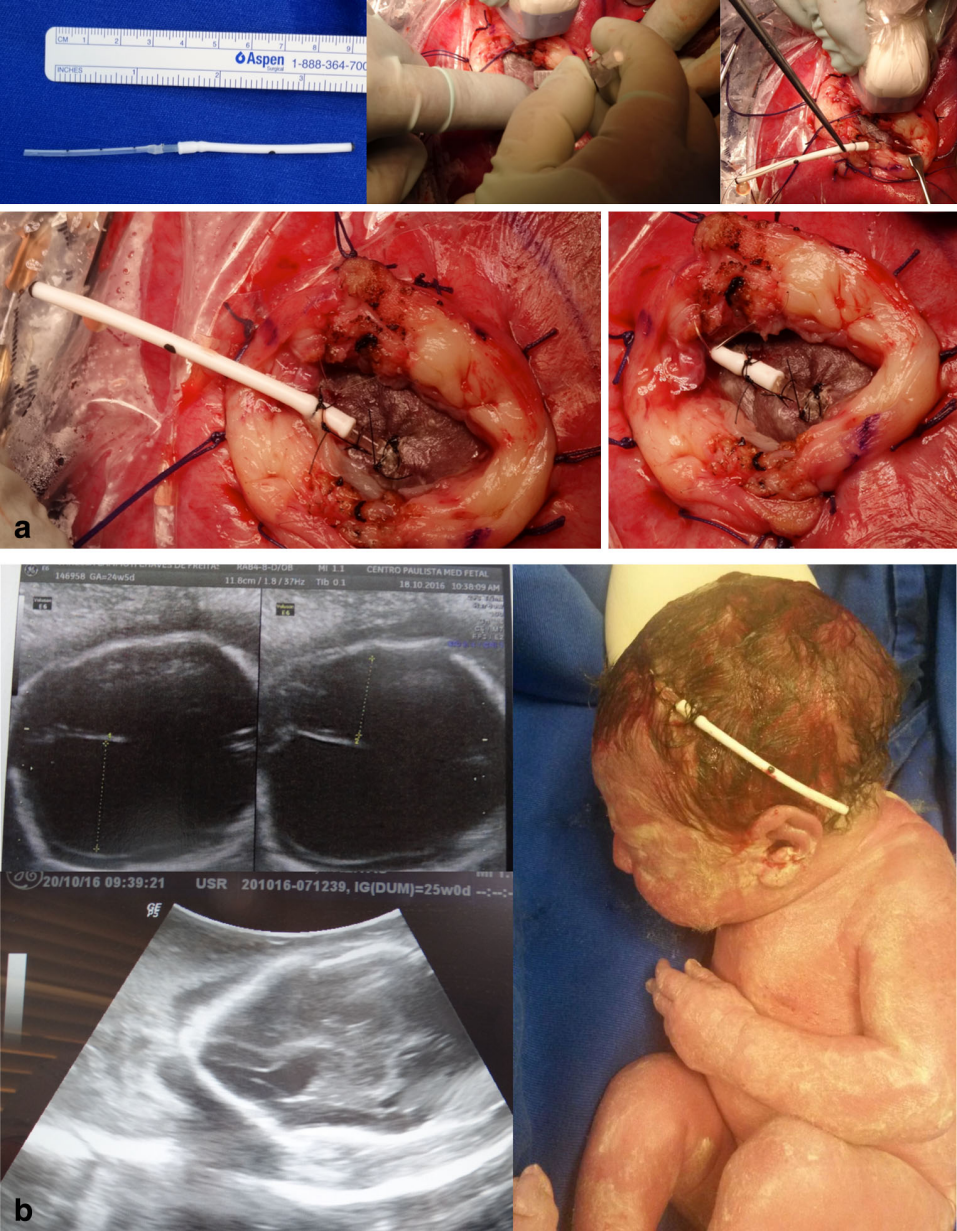
Illustrative case of simultaneous MMC correction and ventriculoamniotic shunt. **a** Insertion of the ventricular catheter with peel-away technique (Valve type Accu-flo Codman® low pressure). **b** Pre- and postoperative ultrasound evidencing the catheter insertion and complete reduction of the ventricle cavity. Neonate picture at the 36th week of pregnancy showing the shunt still working at a good place

Surgery is also not recommended in cases with kyphoscoliosis and injury above L1. Ventriculomegaly greater than 16 mm is a formal MOMS exclusion criterion. However, our cases with ventriculomegaly superior to 20 mm are contraindicated. Many patients have colpocephaly but not real hydrocephalus with macrocrania. Five patients in our series had ventriculomegalia greater than 16 mm, and in one case, there was need for a ventricular shunt after birth. Another that presented with severe hydrocephalus, we corrected the myelomeningocele and placed a ventricle-amniotic shunt (Accu-Flo low rage pressure Codman®) (Fig. 8). We have been a little more flexible about gestational age and have operated on fetuses that are younger than 27 weeks of gestation. Age and weight are other important factors. We have operated on patients under 18 years of age but with the informed consent of parents. Moreover, for some patients with a body mass index greater than 35, we have also recommended surgery.

## Surgical technique

With the promising results of the MOMS trial, the in utero surgical procedure was no longer considered experimental and became available in several centers of fetal medicine with the recommendation of the American College of Obstetrics and Gynecology (ACOG) 2013 [38, 39]. Fetal surgery requires the work of a multidisciplinary team (obstetrician, neurosurgeon, neonatologist, geneticist, anesthesiologist, nurse, physiotherapist, psychologist, and nutritionist, among others, where each professional has a role and is interacting at all times of the treatment).

Based on our initial experience in the monitoring of 6 patients that were operated on at the Paulista School of Medicine/Federal University of Sao Paulo in 2003 (after Professors Joseph Bruner and Noel Tulipan at Vanderbilt University trained the surgical team), we were able to restart the program of prenatal treatment of MM soon after the publication of the MOMS trial. Our protocol follows the same recommendations established by the MOMS trial group with regard to the criteria for screening patients, preoperative evaluation protocol, and postoperative care, with an exception for the opening and closure of the uterus due to the legal impediment in our country to be unable to use a uterine stapler without being licensed by a governmental agency. This impediment forced us to develop an alternative surgical technique.

**Fig. 9:**
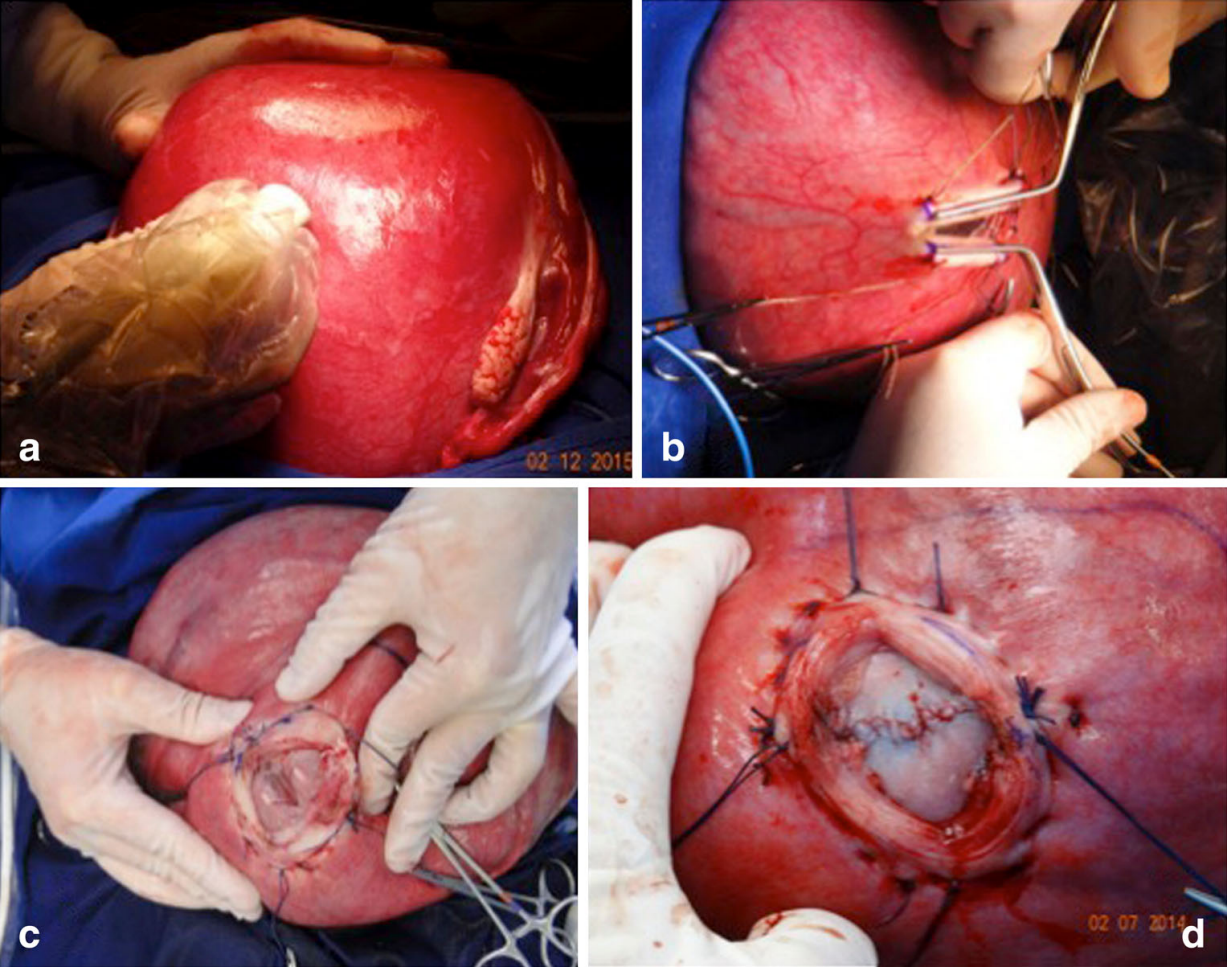
Intraoperative view of open fetal surgery for myelomeningocele. **a** Intraoperative ultrasound monitoring. **b** Cardiac forceps for the uterus hemostasis during its opening. **c** Exposure of the myelomeningocele. **d** Intraoperative view after the myelomeningocele closure

The technique that we use involves opening the abdominal wall (extended Pfannenstiel type incision) and removing the uterus from the abdominal cavity, followed by a detailed mapping of the placental location and identification of fetal and umbilical cord parts by using intraoperative ultrasonography. The location of the hysterotomy will depend on the location of the placenta. When the insertion of the placenta is posterior, we perform an anterior hysterotomy; however, when the placenta is located anteriorly, we conduct a reversal of the surface of the uterus and hysterotomy is performed on the posterior wall of the uterus. The uterus is covered with a sterile plastic bag to prevent heat loss. The temperature of the uterus is monitored continuously with a digital infrared laser thermometer. Six repair sutures are placed on the uterine wall with Vicryl 0 (Fig. 9). A median longitudinal hysterotomy of approximately 5 cm is performed in the uterine wall contralateral to placental insertion using electrocautery and De Bakey vascular clamps. Part of the amniotic fluid is removed and sent to the molecular biology laboratory. The dorsal region of the fetus is positioned at the site of the uterine opening and the neurosurgeon performs the corrective procedure of spina bifida. The temperature of the fetus during fetal surgical procedure is also constantly monitored. After correction of dysraphism, the fetus is released into the uterine cavity. The uterine closure is performed in three steps. The first involves continuous suture of the amniotic membrane and myometrium with monocryl 4–0. The second step involves continuous suture of the myometrium with Vicryl 2–0. The third step involves interrupted sutures of the myometrium with Vicryl 0. Before the complete closure of the uterine wall, a silicone urinary catheter number 10 is inserted into the uterine cavity and the uterus is filled with saline solution at a temperature of 36.8 °C. The laparotomy is closed following the classic patterns for cesarean section.

## Neurosurgical aspects

An invasive procedure in the fetus can be performed as long as some precautions are taken, such as the establishment of an environment with few physiological changes. Silence in the surgical room is also important. The surgery and manipulation of the fetus should occur in the shortest possible time. Our neurosurgical procedures last from 27 to 50 min. On average, we spend 40 min for correction of the spinal dysraphism. A warm environment is also critical. Thus, fetal temperature is continuously remotely monitored during the entire procedure with a digital infrared laser thermometer. Initially, fetuses were anesthetized with an intramuscular dose of fentanyl; however, this strategy was modified after the observation that the mother’s anesthetic is sufficient to anesthetize the fetus. With this change, we no longer observed fetal bradycardia in association with anesthesia.

**Fig. 10:**
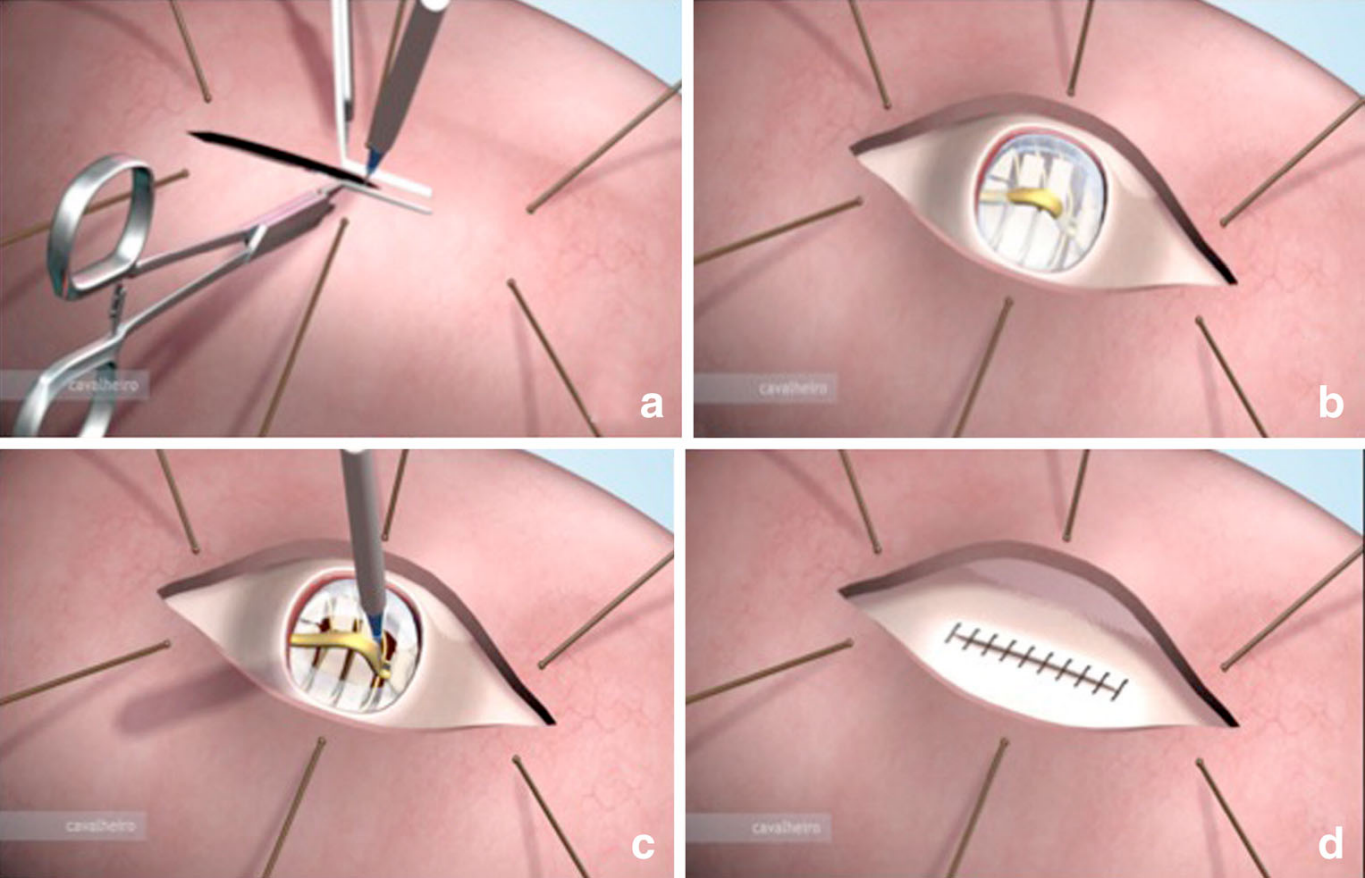
Illustration of the uterus opening. **a** Using electric cautery and hemostatic forceps. **b** Exposure of the myelomeningocele. **c** Cutting the apical ligament producing spinal cord detachment. **d** Final aspect of skin closure

The obstetrician performs a hysterotomy of approximately 5 cm. After the hysterotomy, the fetus is positioned so that the neurosurgeon performs the correction of the MM. The surgical technique employed for the treatment of the MM involves the release of the placode and lumbar and sacral roots because the cord is tethered in this disease. The most important steps are the release of the cord and the treatment of the tethered spinal cord. Often, we find a fibrotic band fixing the top part of the placode to the dura mater, which is what we call “cava” ligament. This ligament was found in more than 90% of cases of MM. Some authors recommend using artificial grafts to close the dura mater without the release of the medulla [40]. This procedure is intended to only protect the neural tissue from the amniotic fluid and intrauterine trauma. For MM, a tethered cord is present from the beginning, and we advocate the release of the cord as one of the most important steps in the procedure. After reconstruction of the placode to its original form, we hermetically close the dura mater with poliglactin 910 (Vicryl) 5.0. Thereafter, a detachment of the aponeurosis with hermetic closing is also performed. In most cases, the dura mater is firmly adhered to the aponeurosis. Thus, we close the two membranes together. Finally, we close the skin with an absorbable suture and a continuous suture with polyglecaprone 25 (monocryl) 5.0 (Fig. 10). In all cases, the skin could be closed, and the healing process was efficient without the use of exogenous material. The neurosurgical procedures were performed with the aid of a Zeiss surgical microscope and/or magnifying glass. After closure of the MM, the fetus is released into the uterine cavity. The amniotic fluid is replaced and the uterus is closed.

In the postoperative period, fetal motility is constantly monitored. In addition to the vital fetal data, the scar of the MM, the size of the ventricular cavity, and the degree of herniation of the brain stem and the cerebellar tonsils are evaluated. The neurosurgical results were considered excellent. The need for ventricular shunts for hydrocephalus was greatly reduced. In our practice, when the surgery is performed after birth, in 90% of cases, it is necessary to place shunts. The study group indicated the need for shunts in 82% of the cases that were operated upon postnatally compared to 40% of cases operated upon in utero. In our series of intrauterine repair, the need for placing shunts was only 7% when the MM was corrected in utero. No patient presented symptoms of Chiari type II, such as respiratory difficulty and laryngeal stridor. The motor level was very much improved. In 42% of the cases, there was a motor improvement of two levels, and 36% presented an improvement of one level. In 22% of the cases, there was no variation between the anatomical and motor level of the lesion. The operative mortality was 1.8% of the cases, and all deaths occurred at the beginning of the series. In the last 2 years, no patient died. Prematurity is still a serious problem and the mean birth is at 34 weeks.

## Postoperative care

Fetal neurosurgery entails the monitoring of the patient fetus from the moment of birth to the postnatal period. It is clear that the more delayed the delivery, the better the conditions of the newborn, but maternal health should be taken into consideration, particularly the quantity of the amniotic fluid and the thickness of the surgical scar. The rupture of amniotic membranes and the transition to oligohydramnios is indicative of the anticipation of labor, as well as the thinning of the uterine scar. If a thinning of the uterine scar is detected, early childbirth can prevent uterine rupture.

In our series of 220 patients who underwent surgery for correction of MM in utero, gestational age ranged from 26 to 37 2/7 weeks. Eighteen cases were born with less than 30 weeks of gestation, 74 cases were born between 30 and 34 weeks, 80 cases were born between 35 and 36 weeks, and 48 cases were born after 37 weeks. Perinatal mortality occurred in 4 cases (1.8%), 2 of which were due to chorioamnionitis. One case of mortality was due to a premature rupture of membranes and another was due to idiopathic bradycardia. This mortality rate is lower than the results of the MOMS trial group (2.5%) and the Children’s Hospital of Philadelphia (CHOP) trials (6%) [41].

**Fig. 11:**
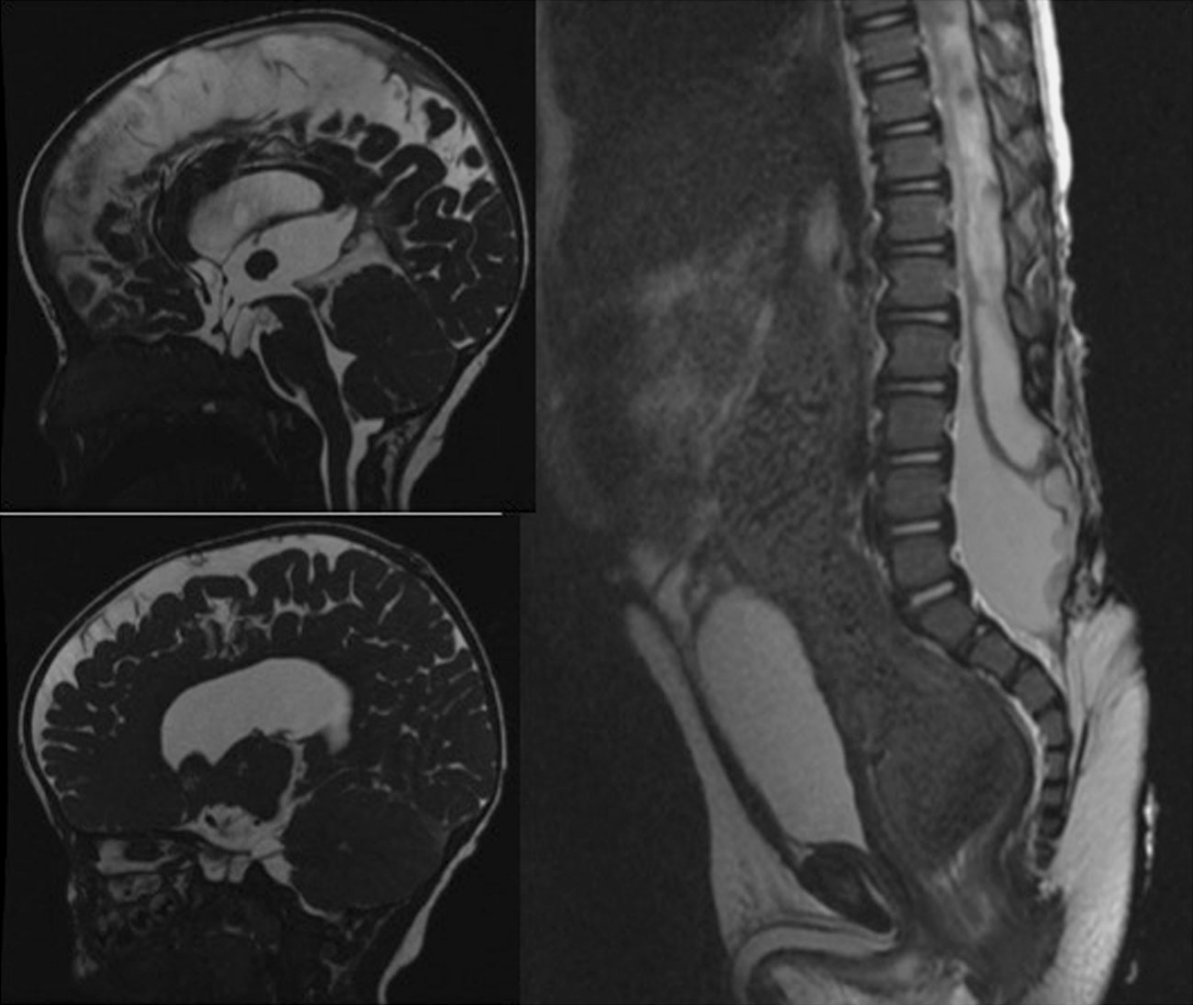
T2-WI MRI of the lumbosacral and skull in a male patient that is 8 months—age that underwent a fetal surgery at 25 weeks of pregnancy showing an absence of intracranial hypertension, patency of the aqueduct, cerebellum in the normal position, and subdural cerebrospinal fluid effusion as a benign hydrocephalus. In the sagittal view is represented a large spinal dysraphism from L2 to S4, showing syringomyelia, tethered cord, and epidermoid tumor

The mean birth weight in our service was lower than that of the MOMS trial and CHOP trials. On average, our newborns weighed 2199 ± 571 g, while in the MOMS and the CHP trials, the average weight of the newborns was 2383 ± 688 and 2415 g, respectively. Fourteen patients required a ventriculoperitoneal shunt up to 5 years later (6.3%), while the MOMS trial placed a shunt in 40% of their cases (31 cases). The majority of cases required a shunt while still in the maternity (10 cases) and were all newborns born before 34 weeks. Significant changes were observed in the degree of hindbrain herniation. In the MOMS trial, 36% did not present herniation, 40% presented a mild herniation, 19% showed a moderate herniation, and 6% showed a severe herniation. In our series, 80% of the cases did not present herniation, 14% showed a mild herniation, and 6% showed a moderate herniation. We had no cases of symptomatic Chiari. There was also an improvement of motor function when compared with the anatomical level. In the MOMS trial, 50% of the patients improved by one or two levels, 23% remained unchanged, and 34% worsened. In our case series, 75% showed improvement, 20% remained unchanged, and 15% worsened. The results of the CHOP trial were different. That is, 55% of the patients improved, 32.5% remained unchanged, and 14% worsened. The motor function is very difficult to evaluate when comparing the anatomical with the motor levels. We did observe that rachischisis presented a better development than the myelomeningocele. Two patients needed to be submitted to reoperation using a tethered cord, and in both cases, the presence of epidermoid tumor was identified. One of the patients required three procedures and presented an extensive MM from L2 to S2 (Fig. 11).

Antenatal MM repair reduces shunt placement rates and improves motor function outcomes; however, an improvement was not observed for bladder function. Clayton et al. [42] reported that 23 (82%) patients in a cohort of 28 used clean intermittent catheterization to manage the bladder at a mean age of 9.6 years, 24 (84%) required a bowel regimen to manage constipation, and 6 (21%) patients underwent lower urinary tract reconstruction. Lee et al. [43] conducted a retrospective study to compare lower urinary tract function between the pre and postnatal MM repair groups and did not find differences between the two. Leal da Cruz et al. [44] analyzed our cohort of the first 51 patients and found that 93.7% had significantly less urinary tract dysfunction of high bladder pressure or incontinence. These results reveal the challenges that still have to be overcome in patients with MM.

## Comparison of fetal and postnatal surgeries

**Fig. 12:**
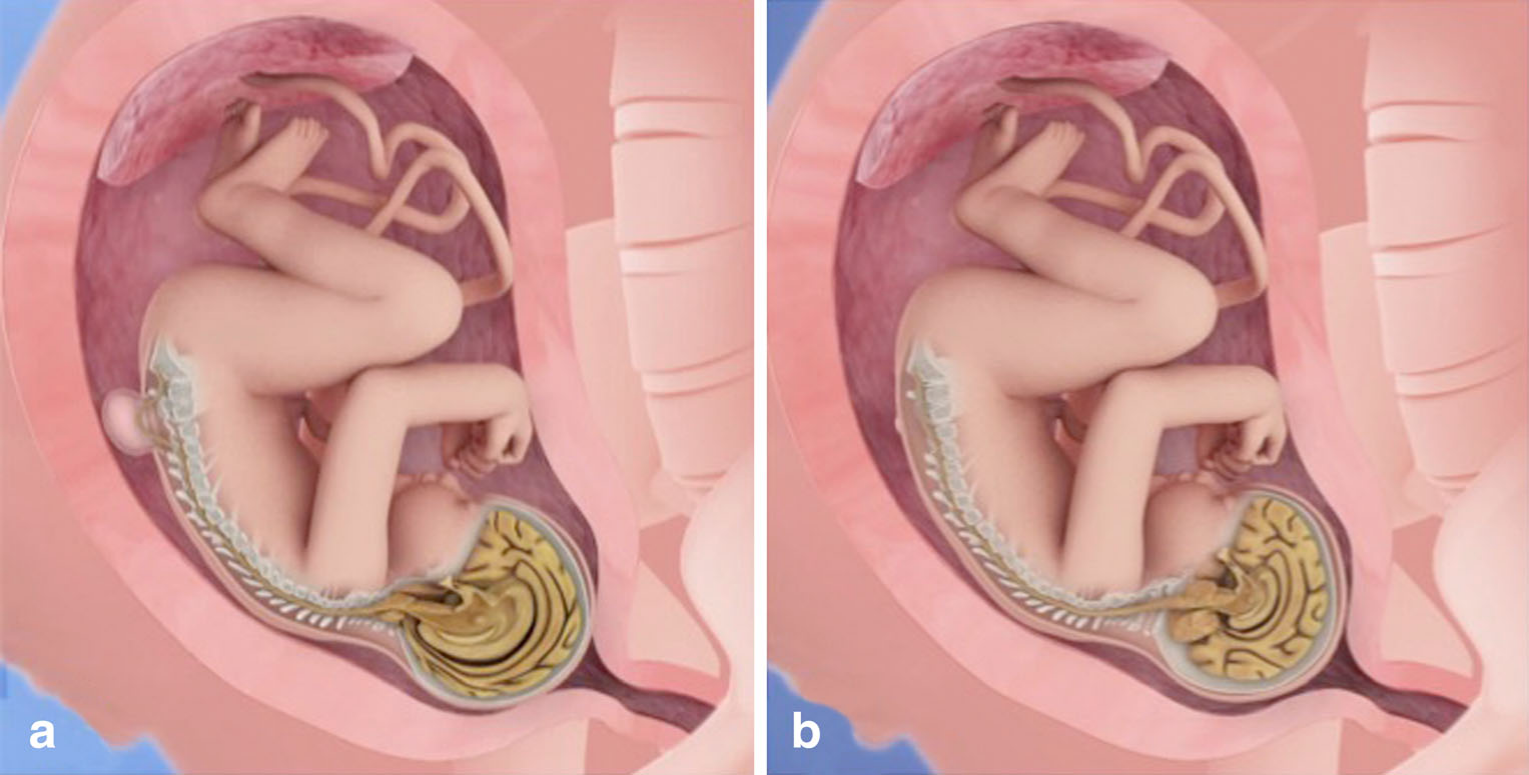
**a** Illustration of the complex myelomeningocele and Chiari type II. **b** Involution of the Chiari type II due to the expansion of the posterior fossa

**Fig. 13:**
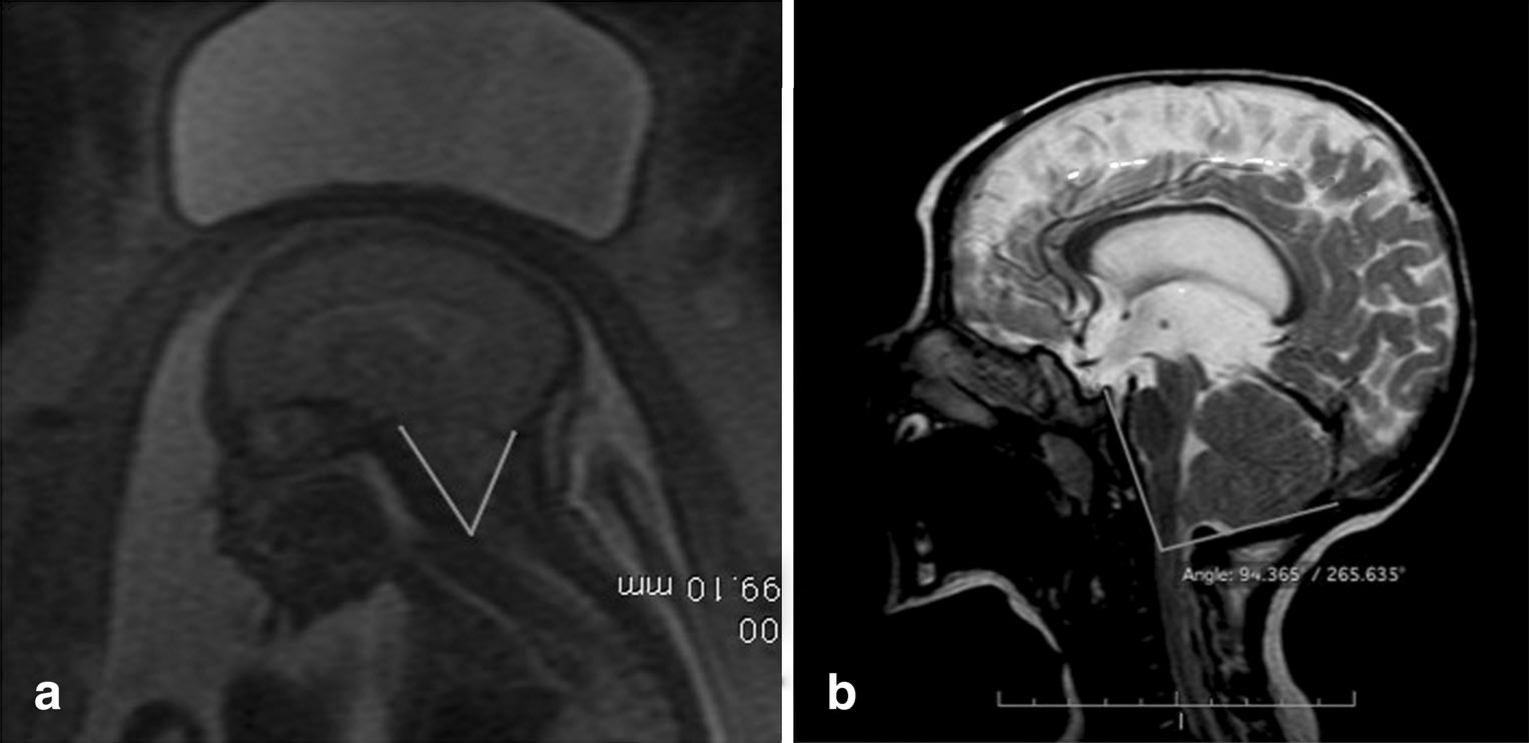
**a** Preoperative MRI showing the fetal clivus-supraocciput angle before the myelomeningocele correction. **b** Opening of the clivus-supraocciput angle after the myelomeningocele correction

The UNIFIED theory proposed by McLone and Knepper (1989) to explain the pathophysiology of Chiari II malformation is sufficient to understand the mechanisms involved in the early and late treatment of MM. Some of these changes can be explained during the fetal surgical procedures but cannot be verified in postnatal surgical procedures. These changes include the “dry brain” that is verified by the lemon sonographic sign and features intracranial hypotension. Another alteration is the presence of a fibrous ligament in the apex of the placode adhered to the dura mater in the apical portion of the malformation that results in a tethered cord and pulls the brainstem and cerebellum down and into the rachidian canal resulting in a microcephaly, a small posterior fossa, and poor development of the subarachnoid space of the posterior fossa. With the development of a pregnancy that is associated with intracranial hypotension, the occipital bone, which consists of 8 segments, fuses rapidly, making the posterior fossa inelastic, similar to that in craniosynostosis. When we perform the correction of a MM after birth, the posterior fossa does not expand, and reversal of the Chiari II does not occur. When surgery is performed before 27 weeks, the posterior fossa is still open, and the increased pressure in the posterior fossa near the closure of the cerebrospinal fluid leakage will result in a broad development of the skull with the disappearance of the lemon sign and an increase in the volume of the posterior fossa. The cerebellum assumes its normal position with the disappearance of herniation together with the development of the subarachnoid space of the posterior fossa (Fig. 12), as verified by the clivus-supraocciput angle (CSA). The preoperative CSA averaged 63°, while the postoperative CSA averaged 84° (Fig. 13). This angle changes in the first 15 days after the closure of the MM. In a separate study, [45] demonstrated that the patients with open neural tube defects had a mean CSA of 53.4° and the normal controls patients had a mean CSA of 78°. In spite of these differences, both studies indicate that open neural tube defects result in a leakage of the CSF, and the associated hindbrain herniation results in a small posterior fossa when compared to normal controls. Our patients have the opportunity to close the neural tube defect early, allowing for an enlargement of the posterior fossa. Moreover, this reshaping of the posterior fossa could influence the cerebrospinal fluid dynamics, which would explain the lower rates of the cerebrospinal shunt procedure in antenatal repaired patients. None of our patients was symptomatic for Chiari.

**Fig. 14:**
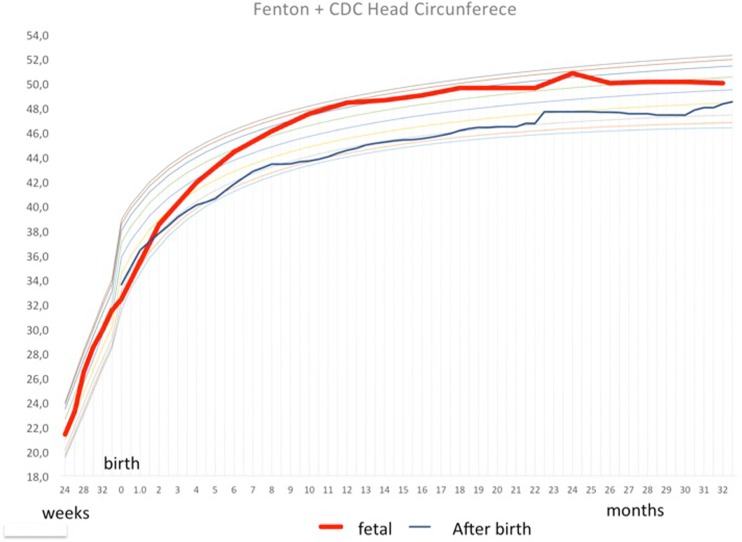
Head circumference curve of patients that was submitted to fetal myelomeningocele correction and followed without ventriculoperitoneal shunt (*red curve*) compared to patients that was submitted to postnatal correction of the myelomeningocele and followed with ventriculoperitoneal shunt (*blue curve*)

Our patients experienced the best outcomes reported thus far. Adzick et al. (2011) [9] reported a shunt placement of 40% in the antenatal corticosteroids group versus 82% in the postnatal group by the age of 12 months. Moreover, they showed that 36% of the patients in the prenatal repair group did not present any degree of hindbrain herniation compared to 4% in the postnatal group. Additionally, Tulipan et al. [46] reported that the main prenatal risk factor for a shunt placement was the ventricle size. That is, 20% of fetuses with a ventricle size <10 mm had a shunt placement compared to 45.2% for those with ventricle size between 10 mm and up to 15 mm and 79% for those with ventricles larger than 15 mm. Johnson et al. [47] reported a shunt placement rate of 42.6% at a mean age of 21.2 weeks and demonstrated that the more cranial the defect, the higher the risk for a shunt placement. In our cohort of 180 patients that were followed for more than 12 months, only 8 patients met the criteria for a shunt placement. These findings differ from the previous reports, mainly because of the use of a different approach to treat hydrocephalus. For those patients who do not develop a bulging fontanelle, split sutures, or a sunset eye sign, we follow the head circumference curve; when the patient crosses two percentiles, we begin to administer acetazolamide at a dose of 250 mg/day and follow the head circumference growth on a monthly basis. We realized that in many patients, the head stops growing and remains stable in the upper percentiles as demonstrated in the curve below (Fig. 14). In these patients, an obstructive hydrocephalus transforms into a communicating hydrocephalus. In addition, the cerebral aqueduct becomes patent, there is no Chiari type II, and the CSF is circulating; however, globally, there is an accumulation of CSF in the subarachnoid space. This leads to an increase in the head circumference without signs of intracranial hypertension and normal neuropsychomotor development.

## Occipital encephalocele

Encephalocele is a congenital neural tube defect characterized by a median cranial bone cleft defect that results in protrusion of the meninges (meningocele) or of the meninges and neural tissue (encephalocele). Encephalocele has an estimated prevalence of 0.8–2.0 per 10,000 live births [48–50]. One third of patients die from this condition, with 76% of deaths occurring in the first day of life. Half of the patients who live beyond the first day will experience some degree of neurodevelopmental delay [51, 52]. The strongest risk factors for death are hydrocephalus and microcephaly [51, 53].

Advances in imaging technologies have enabled early diagnosis of encephalocele in the antenatal period. Specifically, fetal ultrasound has a screening detection rate of 60–94% for neural tube anomalies [54, 55], and fetal magnetic resonance imaging (MRI) displays anatomical details of the malformation, confirms ultrasonography diagnoses, and identifies possible associated anomalies [55, 56]. Moreover, the results of the Management of Myelomeningocele Study (MOMS), which investigated the benefits of antenatal treatment of myelomeningocele as compared to that of postnatal treatment [9], supported the efficacy of fetal surgery for congenital diseases. Therefore, antenatal repair of occipital encephalocele (OE) may effectively stop the progression of encephalocele sac herniation and result in microcephaly reversion. Experiences with fetal surgery for myelomeningocele correction led us to conclude that Chiari type III cases with microcephaly should be treated prior to 27 weeks of gestation.

The selection criteria for inclusion were gestational age between 19 and 27 weeks and 6 days, maternal age ≥18 years, normal fetal karyotype, microcephaly, and cystic hernia sac. The major exclusion criteria were fetal anomaly unrelated to OE, risk of premature birth (including short cervix and previous preterm delivery), and placental abruption.

## Surgical technique

The first steps of the treatment follow the same technique applied to the fetal surgery for myelomeningocele as described above. Correction of OE began with microscopic magnification. A skin incision was made in the herniated sac, following a neurosurgeon’s review of the fetal MRI for details regarding the contents and anatomical position of the OE; the sac is typically incised at the midline. Electric monopolar cautery should be avoided and hemostasis should occur; therefore, we typically use bipolar forceps at a very low intensity. The meningeal tissue is then exposed and 360° dissection occurred, followed by tissue coagulation with bipolar forceps and cutting with microscissors near the outer table of the occipital bone. It is important to maintain a margin of safety from the meningeal layer to the posterior closure, and 2 cm is typically sufficient. Next, the neural tissue is accessed, and the procedure is repeated; vessels and tissues are coagulated and then cut to reduce the neural tissue bellow the inner table of the occipital bone. The meningeal layer is then closed with running 6-0 polypropylene sutures, and the skin flap is cut to avoid tissue overlap. The inner bone and dura mater are adhered to each other with polypropylene 6-0, an absorbable mini-plate (LactoSorb®). Finally, the subcutaneous tissue is closed with uncolored Vicryl 5-0 and the skin is closed with running 6-0 monocryl suture.

**Fig. 15:**
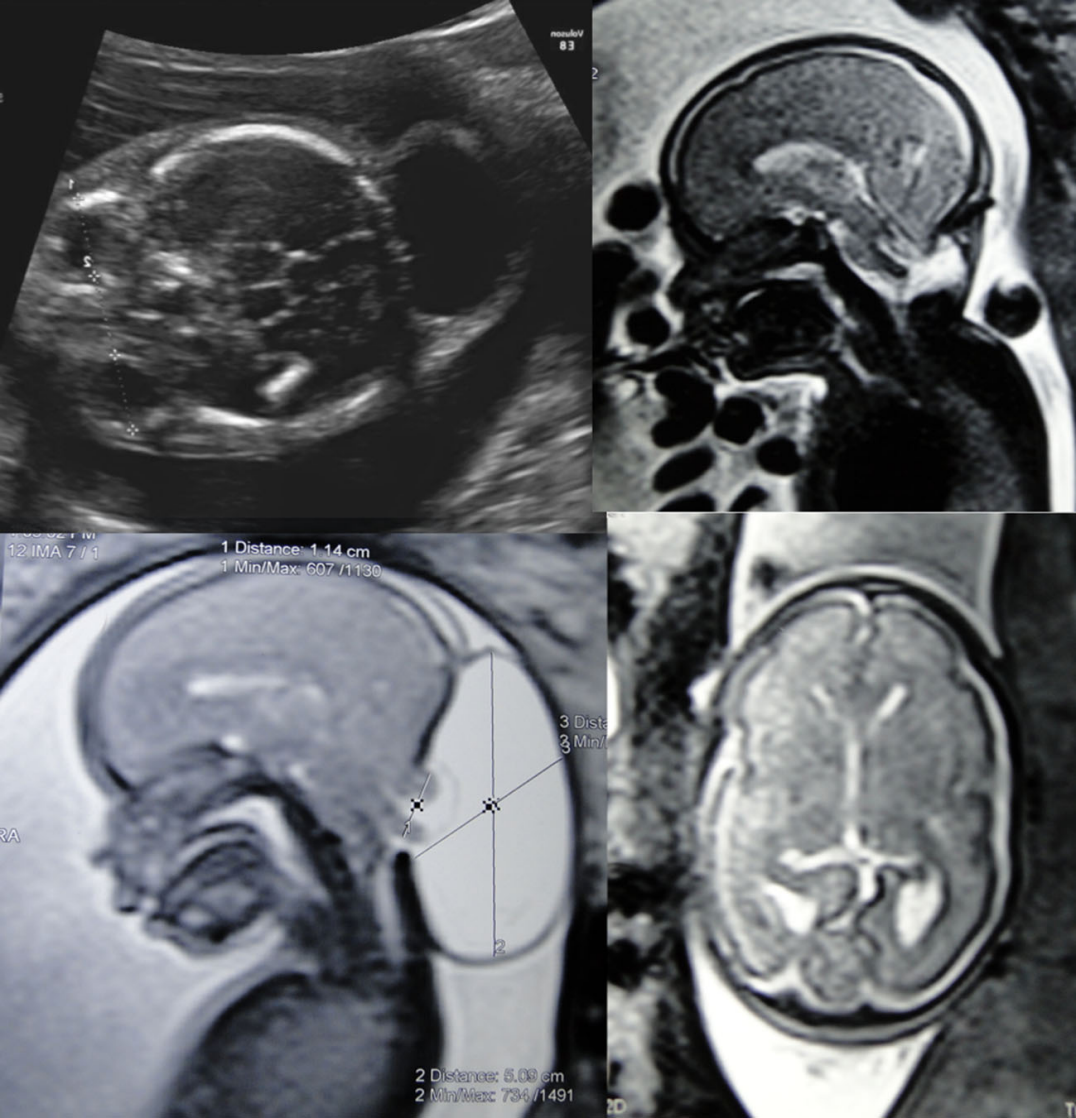
Illustrative case of a fetus that presented occipital encephalocele and trending to microcephaly that underwent to fetal correction at the 26th week of gestation. *Left pictures* illustrate the preoperative ultrasound and MRI evidencing the occipital encephalocele sac. *Right pictures* illustrate the postoperative MRI showing the correction of the Chiari type III

We corrected five patients with occipital encephalocele and microcephaly (Fig. 15). In 4 of the 5 cases, it was possible to place an absorbable plaque between the dura mater and the bone defect. All patients showed a reversal of microcephaly. One patient had preterm labor and her baby was born at 29 weeks of gestation; the baby died of pulmonary problems 10 days later. The other four patients showed an excellent evolution. The average gestational age for the four patients was 36 2/7 weeks. Among the 4 patients, 1 developed hydrocephalus and underwent a ventriculoperitoneal shunt at postnatal day 25. In our literature review, we did not find the description of a treatment for intrauterine occipital encephalocele. We believe that we are the first group to treat this type of disease. It is important to emphasize that the main objective of this intrauterine treatment is the reversal of microcephaly.

### Discussion

With the development of diagnostic methods for identifying fetal neurosurgical diseases, it is crucial that neurosurgeons develop minimally invasive surgical techniques that allow fetuses to benefit from the procedures performed early in the intrauterine life. The main errors made in the treatment of fetal hydrocephalus arise from the difficulty of accurately diagnosing hydrocephalus. It is clear that fetuses with acute obstructive hydrocephalus, which is often caused by a Coxsackie virus infection, benefit from hydrocephalus treatments, while fetuses with chronic destructive ventriculomegaly, such as those seen in Bicker-Adams syndrome or after infection by the Zika virus, will show a catastrophic evolution. Thus, a multicenter cooperative study is required for the treatment of evolutive fetal obstructive hydrocephalus. Further, the inclusion of an efficient surgical technique is also important. Cephalocentesis can be performed safely, as it allows not only a better diagnosis but also an isolated measure of intracranial pressure; it can also be used therapeutically when the fetus reaches lung maturity. We rarely used fetal endoscopic third ventriculostomy due to the difficulty to enter the Kocher’s point to access the third ventricle. However, it is possible to initiate the procedure, and in case the third ventriculostomy cannot be performed, a ventriculoamniotic shunt can be placed. The MOMS results show that it is crucial that several neurosurgery centers are dedicated to this type of treatment during the fetal period. The open surgery technique is the main form of treatment and produces excellent results when compared with those obtained through the traditional technique of closure after birth. Failure can occur during the treatment of fetal myelomeningocele. In most cases, we found a fibrous ligament that attaches the medulla to the dura mater in the upper part of the dysraphism. This ligament is not always found in postnatal procedures, or it is located in cranial positions, which determine the clinical condition of tethered cords. Another phenomenon observed in most cases in the post-operative period is the increase in the posterior fossa volume, which is related to the embryogenesis of the occipital bone and increased intracranial pressure after correction of the fistula.

## Conclusions

Further progress is necessary to enable fetal neurosurgery in becoming the main technique used in treating fetal neurosurgical diseases. However, we believe that correct prenatal diagnosis and adequate selection of fetuses with myelomeningocele, hydrocephalus, and occipital encephalocele may contribute to the benefits provided by neurosurgical procedures during the fetal period.
